# The predictive value of tumor mutation burden for immune checkpoint inhibitors therapy in non-small cell lung cancer is affected by patients’ age

**DOI:** 10.1186/s40364-020-00188-2

**Published:** 2020-04-09

**Authors:** Yongfeng Wu, Jinming Xu, Jiawei Xu, Yiqing Wang, Luming Wang, Wang Lv, Jian Hu

**Affiliations:** 1 Department of Thoracic Surgery, The First Affiliated Hospital, School of Medicine, Zhejiang University, 79 Qingchun Road, Hangzhou, 310003 China; 2 Department of Toxicology, School of Public Health, School of Medicine, Zhejiang University, Hangzhou, 310058 China

**Keywords:** Tumor mutation burden, TMB, Age, Immune checkpoint inhibitor, ICI, NSCLC, Immunosenescence

## Abstract

High tumor mutation burden (TMB), which is associated with increased tumor immunogenicity, has been identified to predict improved response to immune checkpoint inhibitors (ICIs) therapy in non-small cell lung cancer (NSCLC). As host immunity is also significant to eliminate cancer cells, however, its clinical impact on cancer immunotherapy is still largely unknown. Here we explored the influence of age, which is an important characteristic to evaluate immune response of patients, on TMB-based predictive system for ICIs therapy in NSCLC. Our results showed that high TMB was capable of predicting better durable clinical benefit (DCB) in age^low^ group, while it was insignificant in age^high^ group. Besides, the predictive power of TMB for progression-free survival (PFS) and overall survival (OS) was better in age^low^ group than in age^high^ group. Our study illustrated that the predictive value of TMB for ICIs therapy was better in young patients than in elderly patients in NSCLC.

To the Editor,

Tumor mutation burden (TMB) is widely demonstrated to predict the efficacy of immune checkpoint inhibitors (ICIs) in diverse cancers, especially in non-small cell lung cancer (NSCLC) and melanoma [1, 2]. High TMB presents enriched clonal neoantigens and increased tumor immunogenicity, which can improve the response to cancer immunotherapy [3]. However, as host immunity is also significant to eliminate cancer cells, its clinical impact on cancer immunotherapy is still largely unknown. Immunosenescence, which refers to the decline of immune system with aging, may contribute to reduced tumor cell clearance efficiency in body, leading to increased cancer incidence in the elderly [4].

Based on these facts and evidence, we hypothesized that TMB could show better predictive value for cancer immunotherapy in young patients than in elderly patients in NSCLC. In order to test the hypothesis, published clinical data was identified through systematic literature search. Durable clinical benefit (DCB), progression-free survival (PFS) and overall survival (OS) were adopted as endpoints for assessment. Detailed methods were explained in Additional file 1.

We identified three NSCLC immunotherapy cohorts containing 665 patients [1, 5, 6]. Detailed characteristics of patients included were summarized in Additional file 2: Table S1.

Firstly, as was shown in Fig. 1, high TMB was capable of predicting better DCB in age^low^ group. However, the predictive power was insignificant in age^high^ group, indicating high TMB failed to forecast clinical benefit in the group.

**Fig. 1:**
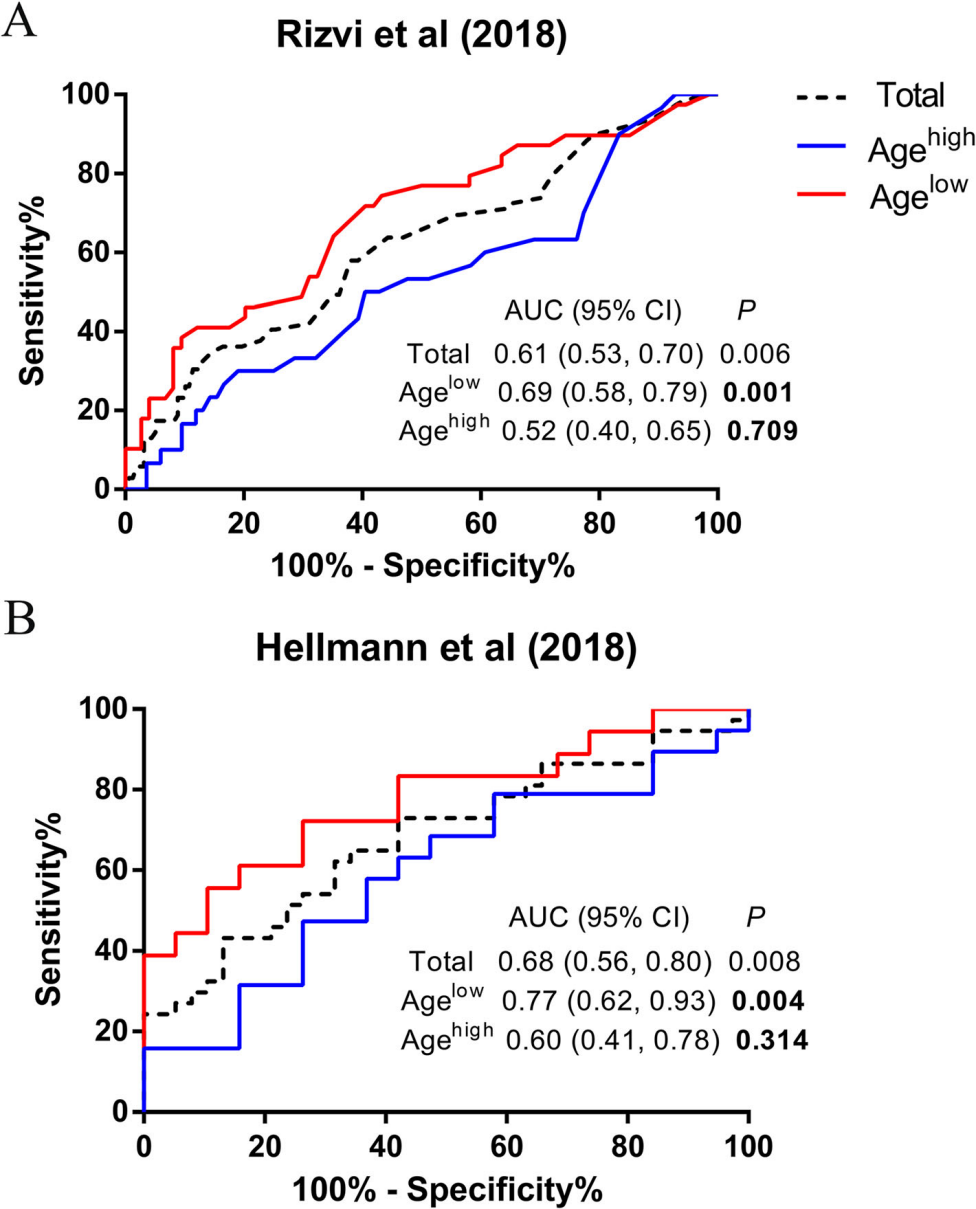
ROC curve analysis of the association between TMB and DCB in young and elderly patients in NSCLC. ROC curves of (**a**) Rizvi cohort, (**b**) Hellmann cohort. ROC: receiver operator characteristic; TMB: tumor mutation burden; DCB: durable clinical benefit; NSCLC: non-small cell lung cancer; AUC: area under curve; CI: confidence interval

Secondly, it was found that in age^low^ group, high TMB dramatically illustrated improved PFS (Rizvi cohort: Hazard ratio [HR] 0.55, 95% confidence interval [CI] 0.35, 0.80, *P* = 0.003, Fig. 2a; Hellman cohort: HR 0.26, 95% CI 0.08, 0.45, *P* < 0.001, Fig. 2c). The results were still significant in multivariate analysis (Rizvi cohort: Adjusted HR 0.54, 95% CI 0.36, 0.82, *P* = 0.004; Hellman cohort: Adjusted HR 0.23, 95% CI 0.09, 0.55, *P* = 0.001). However, there was no correlation between PFS and TMB level in age^high^ group (Rizvi cohort: HR 1.03, 95% CI 0.70, 1.51, *P* = 0.898, Fig. 2b; Hellman cohort: HR 0.71, 95% CI 0.32, 1.55, *P* = 0.388, Fig. 2d). In the adjusted model, the conclusion was unchanged (Rizvi cohort: Adjusted HR 1.10, 95% CI 0.71, 1,71, *P* = 0.677; Hellman cohort: Adjusted HR 0.60, 95% CI 0.24, 1.50, *P* = 0.275). Then, the result of meta-analysis further illustrated that predictive power of TMB was more significant in age^low^ group than in age^high^ group (Heterogeneity between two groups: *P* = 0.007, Fig. 3). In addition, in order to exclude whether the specific cutoff of TMB had an effect on the result, TMB at the highest quarter was adopted as another cutpoint. As was shown in Additional file 2: Figure S1, high TMB still showed better predictive power of PFS in age^low^ group rather than in age^high^ group (Heterogeneity between two groups: *P* = 0.012).

**Fig. 2:**
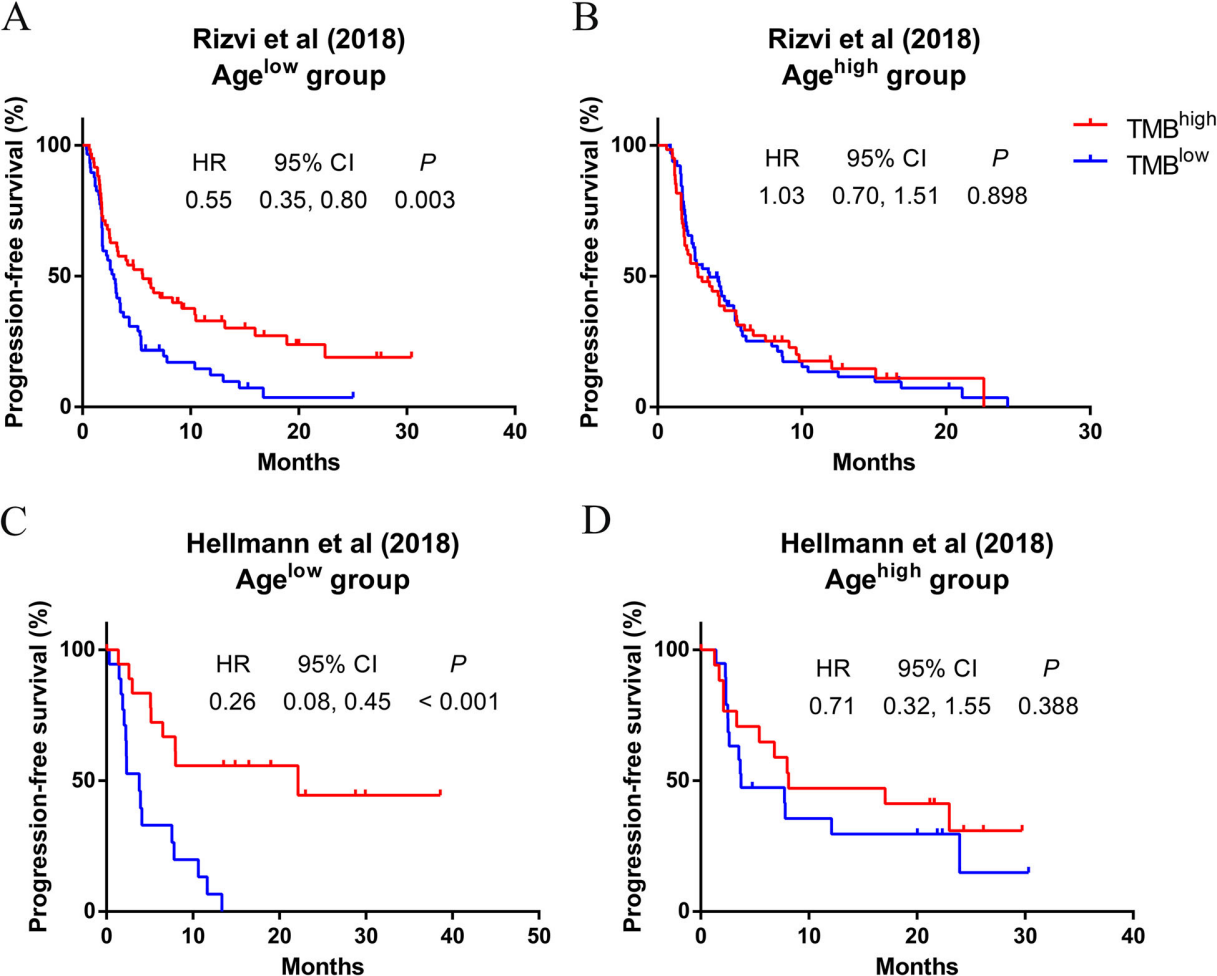
Kaplan–Meier curves and HR analysis of the association between TMB and PFS in young and elderly patients in NSCLC. Kaplan–Meier curves of (**a**) Age^low^ group and (**b**) Age^high^ group in Rizvi cohort, (**c**) Age^low^ group and (**d**) Age^high^ group in Hellmann cohort. HR: hazard ratio; TMB: tumor mutation burden; PFS: progression-free survival; NSCLC: non-small cell lung cancer; CI: confidence interval

**Fig. 3:**
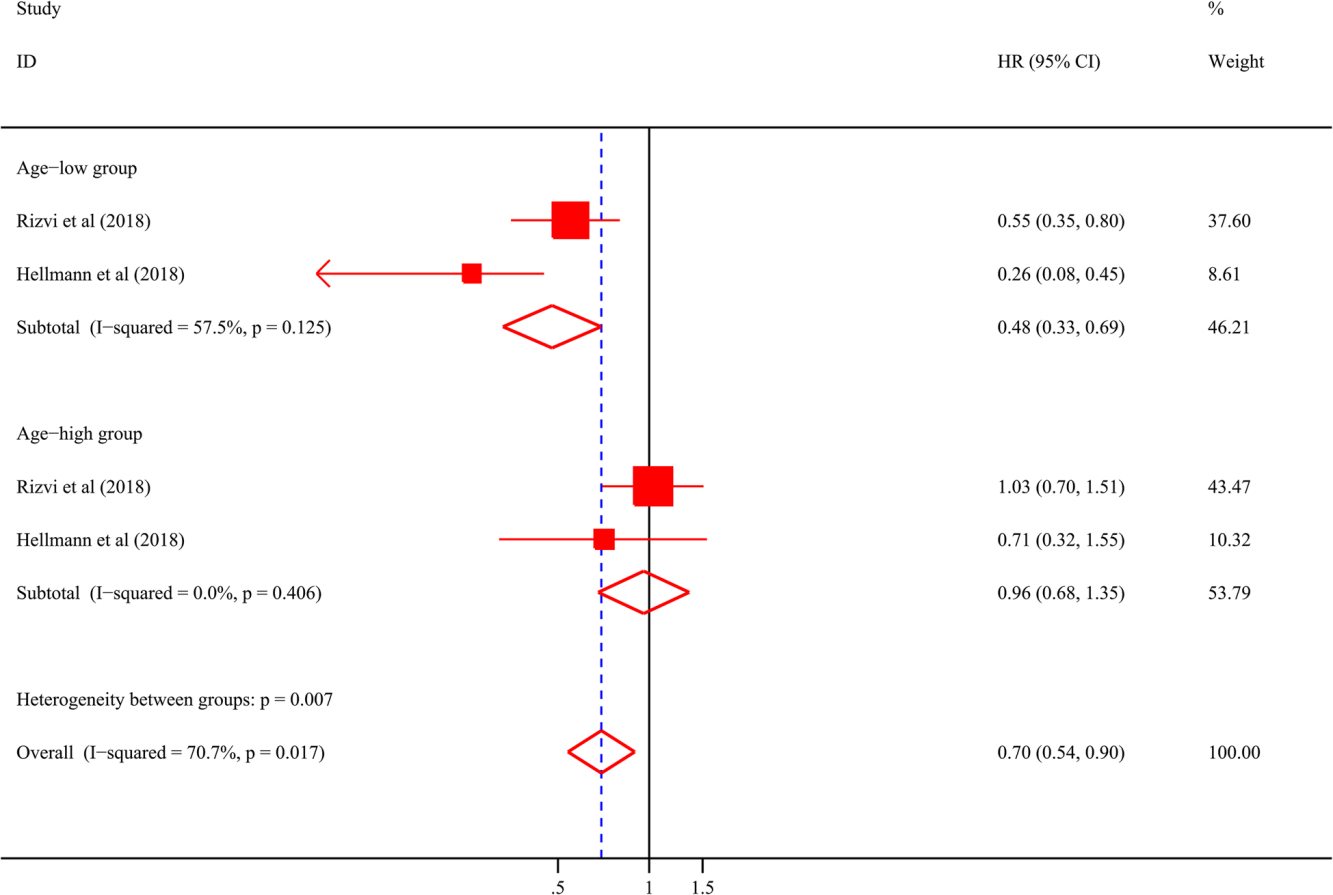
Forest plot of the association between TMB and PFS in young and elderly patients in NSCLC. TMB: tumor mutation burden; PFS: progression-free survival; NSCLC: non-small cell lung cancer; HR: hazard ratio; CI: confidence interval

Moreover, the predictive value of TMB for OS of immunotherapy was evaluated in the two age groups. When using median TMB as cutoff, though both age^low^ (HR 0.72, 95% CI 0.46, 1.07, *P* = 0.112, Additional file 2: Figure S2A) and age^high^ (HR 1.03, 95% CI 0.72, 1.47, *P* = 0.881, Additional file 2: Figure S2B) groups showed insignificant predictive value, the former presented a better tendency. When adopting TMB at the highest quarter as cutpoint, high TMB illustrated meaningful predictive power in age^low^ group (HR 0.43, 95% CI 0.30, 0.76, *P* = 0.007, Additional file 2: Figure S2C), while it was still insignificant in age^high^ group (HR 0.80, 95% CI 0.53, 1.19, *P* = 0.282, Additional file 2: Figure S2D). In multivariate analysis, the conclusion was unchanged (Age^low^ group: Adjusted HR 0.43, 95% CI 0.24, 0.75, *P* = 0.003; Age^high^ group: Adjusted HR 0.82, 95% CI 0.54, 1.25, *P* = 0.354).

In the present study, we found that TMB could present better predictive value on the response to cancer immunotherapy in young patients than in elderly patients in NSCLC. High TMB is associated with enhanced tumor immunogenicity [3], which is an important factor in determining efficacy of cancer immunotherapy [7]. However, in addition to sufficient antigen presentation, a dynamic host immunity may also be necessary for eliminating cancer cells during ICIs therapy. In the process of aging, immune cells are gradually reduced in quantity and becoming defective [4]. Interestingly, PD-1/PD-L1 blockade could not completely restore exhausted T-cell responses in aged mice [8], suggesting the significance of basic immunity of host to confront tumor. To note, age itself may not be an appropriate marker to predict cancer immunotherapy response due to its complex influence on body besides host immunity [9].

There are several strengths in the study. Firstly, different studies including a large number of patients were analyzed, which increased the credibility of the results. Besides, both univariate and multivariate analyses were adopted, which improved the accuracy of the conclusion. However, there are quite a few limitations in the present study. First of all, different TMB testing methodologies and cutoffs were utilized, as there is still no uniform standard, which could lead to heterogeneity of the results. In addition, analysis in the study was conducted only in NSCLC, while related open access data in most cancers are still insufficient.

In conclusion, we revealed that the predictive power of TMB on ICIs therapy was better in young patients than in elderly patients in NSCLC. Therefore, more effective markers need to be identified to differentiate the efficacy of cancer immunotherapy in elderly patients in NSCLC. In addition, the combination of tumor immunogenicity and host immunity evaluation may better identify patient subgroups which are suitable for cancer immunotherapy.

## Supplementary information

**Additional file 1.** Supplementary Methods

**Additional file 2: Table S1.** Clinical characteristics of included NSCLC cohorts treated with immune checkpoint inhibitors. **Figure S1.** Forest plot of the association between TMB (using the highest quarter as cutoff) and PFS in young and elderly patients in NSCLC. **Figure S2.** Kaplan–Meier curves and HR analysis of the association between TMB and OS in young and elderly patients in NSCLC. Kaplan–Meier curves of (A-B) using median TMB as cutoff and (C-D) using the highest quarter as cutoff.

## Data Availability

The datasets analyzed during the current study are available at cBioPortal (Samstein et al. (2019): https://www.cbioportal.org/study/summary?id=tmb_mskcc_2018 [1]; Rizvi et al. (2018): https://www.cbioportal.org/study/summary?id=nsclc_pd1_msk_2018 [5]) and ScienceDirect (Hellmann et al. (2018): 10.1016/j.ccell.2018.03.018 [6]).

## Abbreviations

TMB: Tumor mutation burden
ICIs: Immune checkpoint inhibitors
NSCLC: Non-small cell lung cancer
DCB: Durable clinical benefit
PFS: Progression-free survival
OS: Overall survival
HR: Hazard ratio
CI: Confidence interval
ROC: Receiver operator characteristic
AUC: Area under curve
