# Supplementary material for: The predictive value of tumor mutation burden for immune checkpoint inhibitors therapy in non-small cell lung cancer is affected by patients’ age
Source: Biomark Res. 2020 Apr 9;8:9. doi: 10.1186/s40364-020-00188-2 (PMC7146978; doi:10.1186/s40364-020-00188-2)
Supplement: Supplementary file 1 — Additional file 1. Supplementary Methods [file 40364_2020_188_MOESM1_ESM.pdf]

Supplementary Methods

Literature search and study design

Systematic literature search was conducted on PubMed, EMBASE, Web of Science and Cochrane Library up to December 8, 2019. The detailed search terms were as follows: (PD-1 OR PD-L1 OR CTLA-4 OR Ipilimumab OR Tremelimumab OR Nivolumab OR Pembrolizumab OR Lambrolizumab OR Atezolizumab OR Avelumab OR Durvalumab OR “immune checkpoint inhibitor” OR “immune checkpoint inhibitors” OR “ICI” OR “ICIs” OR “immune checkpoint blocker” OR “immune checkpoint blockers” OR “ICB” OR “ICBs”) AND (mutation burden OR mutational burden OR mutation load OR mutational load OR TMB OR TML). The inclusion criteria were as follows: (1) studies with individual nonsynonymous TMB level, the response to ICIs (DCB, PFS or OS) and age were accessible; (2) the number of patients was more than 50. Clinical characteristics of patients were acquired from articles or cBioPortal (https://www.cbioportal.org/).

In order to compare the association between TMB and efficacy of ICIs in different age groups, patients were divided into age^{high} (≥ 65) and age^{low} (< 65) groups as most studies used (1), as well as TMB^{high} and TMB^{low} groups, and median TMB was adopted as cutoff according to each study. To evaluate the correlation between TMB and DCB in age^{high} and age^{low} groups, ROC curves were conducted, and AUC with its 95% CI was estimated. For survival analysis, Kaplan–Meier curves were drawn, and the log-rank test was adopted in univariate analysis. Besides, cox proportional hazards regression

models were applied for multivariate analyses. To note, smoking status, line of therapy and sex were adjusted in Rizvi cohort (2), and ECOG performance status, smoking status, PD-L1 status and sex were adjusted in Hellmann cohort (3), while only sex was adjusted in Samstein cohort (4) due to the limited information. Moreover, meta-analysis was conducted, as pooled HRs in age^{high} and age^{low} groups were estimated respectively by using inverse variance weighted fixed-effects model, and heterogeneity between the two groups was assessed.

Statistical analysis

Statistical analyses were conducted using GraphPad Prism (version 6.01, GraphPad Software, San Diego, CA, USA), SPSS (version 20.0, IBM Corp, Armonk, NY, USA) and STATA (version 11.0, StataCorp, College Station, TX, USA). Two-tailed test was performed for all analyses, and $P < 0.05$ was considered statistically significant.

References

1. Wu Q, Wang Q, Tang X, Xu R, Zhang L, Chen X, et al. Correlation between patients' age and cancer immunotherapy efficacy. Oncoimmunology. 2019;8(4):e1568810.
2. Rizvi H, Sanchez-Vega F, La K, Chatila W, Jonsson P, Halpenny D, et al. Molecular Determinants of Response to Anti-Programmed Cell Death (PD)-1 and Anti-Programmed Death-Ligand 1 (PD-L1) Blockade in Patients With Non-Small-Cell Lung Cancer Profiled With Targeted Next-Generation Sequencing. J Clin Oncol. 2018;36(7):633-41.

3. Hellmann MD, Nathanson T, Rizvi H, Creelan BC, Sanchez-Vega F, Ahuja A, et al. Genomic Features of Response to Combination Immunotherapy in Patients with Advanced Non-Small-Cell Lung Cancer. Cancer Cell. 2018;33(5):843-52.e4.
4. Samstein RM, Lee C-H, Shoushtari AN, Hellmann MD, Shen R, Janjigian YY, et al. Tumor mutational load predicts survival after immunotherapy across multiple cancer types. Nat Genet. 2019;51(2):202-6.
