# Supplementary material for: The predictive value of tumor mutation burden for immune checkpoint inhibitors therapy in non-small cell lung cancer is affected by patients’ age
Source: Biomark Res. 2020 Apr 9;8:9. doi: 10.1186/s40364-020-00188-2 (PMC7146978; doi:10.1186/s40364-020-00188-2)
Supplement: Supplementary file 2 — Additional file 2: Table S1. Clinical characteristics of included NSCLC cohorts treated with immune checkpoint inhibitors. Figure S1. Forest plot of the association between TMB (using the highest quarter as cutoff) and PFS in young and elderly patients in NSCLC. Figure S2. Kaplan–Meier curves and HR analysis of the association between TMB and OS in young and elderly patients in NSCLC. Kaplan–Meier curves of (A-B) using median TMB as cutoff and (C-D) using the highest quarter as cutoff. [file 40364_2020_188_MOESM2_ESM.pdf]

Table S1. Clinical characteristics of included NSCLC cohorts treated with immune checkpoint inhibitors

	**Rizvi et al (2018)**	**Hellmann et al (2018)**	**Samstein et al (2019)**
**No. of patients**	240	75	350
**Age**			
**Median, yrs**	66	66	67
**Range, yrs**	22-92	42-87	31-90
**Gender**			
**Male**	118 (49%)	37 (49%)	170 (49%)
**Female**	122 (51%)	38 (51%)	180 (51%)
**Sequencing method**	Targeted NGS	WES	Targeted NGS
**Median TMB**	7.38 muts/Mb	158 muts	6.89 muts/Mb
**Endpoints**	DCB, PFS	DCB, PFS	OS

NSCLC: non-small cell lung cancer; No.: number; yrs: years; NGS: next generation sequencing; WES: whole exome sequencing; TMB, tumor mutation burden; muts: mutations; Mb: megabase; DCB: durable clinical benefit; PFS: progression-free survival; OS: overall survival.

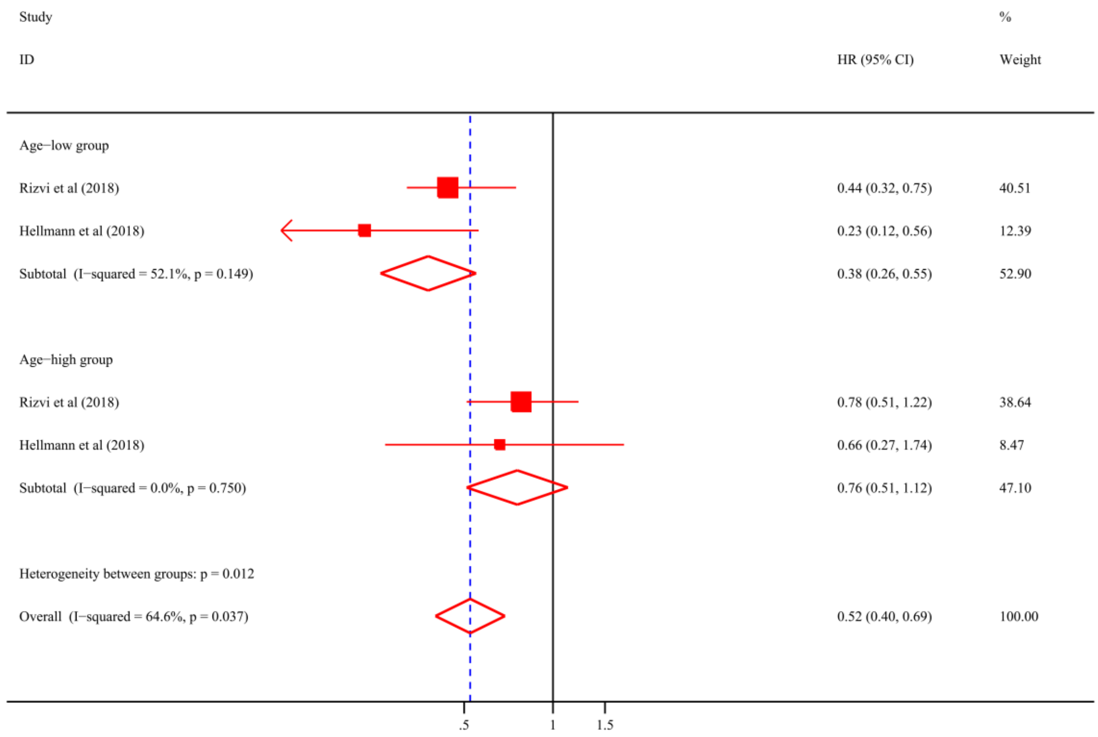

Figure S1. Forest plot of the association between TMB (using the highest quarter as cutoff) and PFS in young and elderly patients in NSCLC. TMB: tumor mutation burden; PFS: progression-free survival; NSCLC: non-small cell lung cancer; HR: hazard ratio; CI: confidence interval.

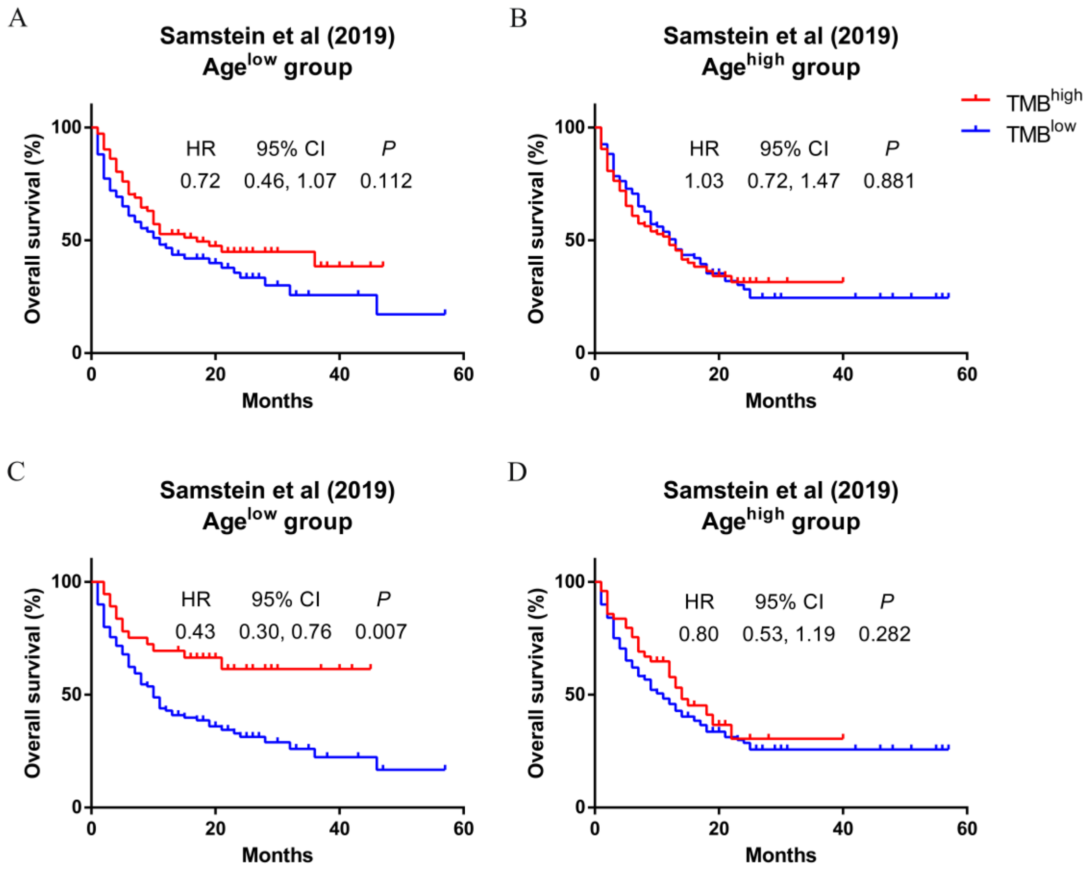

Figure S2. Kaplan–Meier curves and HR analysis of the association between TMB and OS in young and elderly patients in NSCLC. Kaplan–Meier curves of (A-B) using median TMB as cutoff and (C-D) using the highest quarter as cutoff. HR: hazard ratio; TMB: tumor mutation burden; OS: overall survival; NSCLC: non-small cell lung cancer. CI: confidence interval.
